# RNAi strategies to silence HIV-1

**DOI:** 10.1186/1742-4690-8-S2-O34

**Published:** 2011-10-03

**Authors:** Julia Eekels, Veronica lannuci, Rienk E Jeeninga, Bruno Verhasselt, Ben Berkhout

**Affiliations:** 1 Laboratory of Experimental Virology, University of Amsterdam, Meibergdreef 15, 1105 AZ Amsterdam, The Netherlands; 2 Department of Clinical Chemistry, Microbiology and Immunology, Ghent University, De Pintelaan 185, 9000 Gent, Belgium

## 

RNAi is a sequence-specific gene silencing mechanism induced by double-stranded RNA and a promising tool for the development of a durable gene therapy against HIV-1. Such RNAi attack usually focuses on targets within the HIV-1 RNA genome. Despite potent inhibition, we previously observed the rapid selection of RNAi-resistant HIV-1 variants with a mutated target sequence. A number of strategies were tested to prevent viral escape. Similar to the success of combination therapy with multiple anti-retroviral drugs, one can target HIV-1 with multiple short hairpin RNAs (shRNAs). Alternatively, one can design second-generation shRNAs to target favorite RNAi-resistance mutations in order to block specific viral escape routes. One could also combine RNAi with regular antiviral drug regimens. Another strategy involves the use of RNAi against cellular proteins that are involved in HIV-1 replication. The idea is that viral escape is more difficult in this setting. Many cellular co-factors have been described in literature, e.g. based on large RNAi-screens in cell lines. We have shown that long-term inhibition of HIV-1 replication is possible with RNAi against cellular co-factors. To identify relevant co-factors that are not essential for cellular function, primary cells are to be preferred over T cell lines. We propose that RNAi against both viral and cellular targets could be used as a new therapeutic approach in the combat against HIV-1 infection.

